# pH profiling reveals progressive wound acidification during healing and higher pH in chronic non-healing wounds: a prospective, multicenter cohort study

**DOI:** 10.1038/s41598-026-45000-7

**Published:** 2026-03-27

**Authors:** Julian-Dario Rembe, Mareike Witte, Neslihan Ertas, Joachim Dissemond, Waseem Garabet, Maria Hovhannisyan, Katharina Beckamp, Hubert Schelzig, Markus U. Wagenhäuser, Ewa K. Stuermer

**Affiliations:** 1 Department of Vascular and Endovascular Surgery, University Hospital Düsseldorf, Heinrich-Heine-University Düsseldorf (HHU), Moorenstrasse 5, 40225 Düsseldorf, Germany; 2 Department of Dermatology, Venereology and Allergology, Essen University Hospital, Essen, Germany; 3 Clinic and Polyclinic for Vascular Medicine, University Heart and Vascular Center, University Medical Center Hamburg-Eppendorf (UKE), Hamburg, Germany

**Keywords:** pH, Wound temperature, Wound healing, Chronic wound, Wound infection, Wound micro-environment, Biochemistry, Biomarkers, Diseases, Medical research

## Abstract

The local microenvironment of wounds plays a crucial role in healing, with pH and temperature emerging as promising biomarkers. This prospective, multi-center, observational study assessed pH and temperature in acute and chronic wounds to identify physicochemical patterns associated with healing. A total of 117 patients with various wound etiologies underwent 226 pH and 181 temperature measurements at the wound center, edge, and surrounding skin. With a pH of 7.113 ± 0.659 and 7.043 ± 0.694, chronic and non-healing wounds exhibited significantly higher pH values than acute (6.592 ± 0.617) and healing wounds (6.820 ± 0.814; *p*<.05), with the wound center showing the largest difference (Δ_pH_ = 0.52). A pH gradient was observed, decreasing from wound center to surrounding skin. Longitudinal analysis revealed a weekly pH decline of 0.05 units in healing wounds. Temperature showed a similar downward trend over time, though differences between groups were less pronounced. These findings indicate that progressive acidification of the wound environment correlates with healing, while alkaline pH is associated with chronicity and infection. Local pH, more so than temperature, may serve as a non-invasive, dynamic biomarker for wound monitoring and as a potential therapeutic target. Further interventional studies are warranted to evaluate pH-monitoring and potential modulation by wound therapies.

## Introduction

Wound healing is a highly regulated biological process involving distinct but overlapping phases of inflammation, proliferation, and remodeling^1^. While acute wounds, e.g. caused by trauma or surgery, typically proceed through this sequence uneventfully, chronic wounds are characterized by persistent inflammation and failure to progress towards tissue regeneration, despite appropriate clinical management^2^. These chronic wounds are often associated with vascular disease, diabetes, immobility, or infection^2,3^, and their rising prevalence constitutes a growing burden on healthcare systems worldwide^4^.

In recent years, growing attention has been directed toward the physicochemical environment of wounds, particularly local pH and temperature, as potential modulators and biomarkers of healing progression^5–8^. The physiological pH of intact human skin ranges between 5.4 and 5.9, creating an acidic barrier that supports barrier function and antimicrobial defense^9,10^. Upon skin injury, this acidic environment is disrupted, exposing deeper tissues with a near-neutral pH of around 7.4. Chronic wounds, especially when colonized or infected, often exhibit an alkalized microenvironment with pH values reported between 7.2 and 8.9^11,12^. This shift may impair key regenerative processes including fibroblast activity, collagen synthesis, angiogenesis, and protease regulation, which are known to operate optimally in slightly acidic conditions^7,13^.

The pH milieu also affects microbial behavior. Alkaline environments favor the growth of pathogenic species such as *Pseudomonas aeruginosa* and *Staphylococcus aureus*, facilitate biofilm formation, and potentially diminish the efficacy of antibiotics^8,14^. Conversely, mildly acidic conditions appear to inhibit microbial colonization and promote tissue repair, making therapeutic acidification a potential strategy in chronic wound management^15,16^. However, the exact pH range that optimally supports healing remains to be defined and may vary by wound type and phase.

Temperature is another critical but understudied parameter in wound physiology. It affects enzymatic activity, tissue perfusion, oxygen tension, and immune response. Elevated temperatures during the early inflammatory phase of acute wounds are associated with enhanced perfusion and cellular activity, while low temperatures—such as those resulting from peripheral ischemia or perioperative hypothermia—can delay healing. In contrast, chronic wounds may present elevated local temperatures as a sign of ongoing inflammation or infection^17–19^. Previous studies report wound bed temperatures ranging from 31 to 35 °C in chronic ulcers, although precise thresholds for optimal or pathological ranges remain undefined^17,18^.

Despite these findings, few studies have systematically assessed both pH and temperature across different wound types and healing stages. Moreover, data correlating these physicochemical parameters with clinical assessment of wound healing remain limited and heterogeneous. This study aims to investigate the local pH and temperature profiles in a range of acute and chronic wounds. By correlating these measurements with clinical assessments of wound type and healing phase, we aim to identify patterns and potential ranges that distinguish between healing and non-healing wounds. A deeper understanding of how pH and temperature reflect or influence the healing trajectory in both acute and chronic wounds could support the use of pH and temperature as diagnostic or therapeutic parameters in modern wound care.

## Materials and methods

### Study design and patient recruitment

The study was designed as a prospective, longitudinal, epidemiological observational study that was conducted at several clinical centers. Patients were recruited at Helios University Hospital Wuppertal (HUKW), University Hospital Essen (UKE) and University Hospital Düsseldorf (UKD) between January 2019 and December 2022.

The study included patients aged 18 years and older who had either an acute or chronic wound. Chronic wounds were defined in accordance with the standards defined by the German professional medical association Initiative Chronische Wunde e.V. (ICW) as wounds that had existed for more than 8 weeks without clinical healing tendency under previously adequate therapy or were caused by an underlying disease considered chronic, such as diabetic foot ulcers, venous or arterial ulcerations and pressure ulcers^20^. Acute wounds existed for at least 24 h and no longer than 8 weeks after trauma or surgical intervention.

The following aspects were considered exclusion criteria:

- Minors (age < 18 years).
- Presence of dry necrosis.
- Wound area of < 1.5 cm^2^.
- Pregnancy and breastfeeding.
- Malignant origin of the wound.
- Lack of capacity to consent or inability to consent.

Measurements were taken at irregular intervals during scheduled routine check-up visits as part of medical wound therapy. Specific study visits did not take place due to the chosen observational, non-interventional study design, so there was no structured follow-up. The number of measurements taken per patient therefore varies. Additional metadata on demographics, progression and therapy was collected from the study participants (e.g. patient age, gender, wound type, entity, size, duration, stage, healing phase and tendency).

Since the study was designed as a prospective, non-interventional observational cohort study, no standardized microbiological wound swabs or pathogen-specific laboratory testing were mandated by the study protocol. Microbiological sampling was performed only when clinically indicated as part of routine patient care at the discretion of the treating health-care professional (HCP). Consequently, microbiological culture results were available only inconsistently and were not systematically collected for all wounds.

For study purposes, wound colonization status was determined based on structured clinical assessment by the treating HCP. In accordance with the International Wound Infection Institute (IWII) wound infection continuum, all wounds were considered at least contaminated. Clinical signs suggestive of colonization included the presence of visible slough, retained necrotic tissue or colored wound coatings. These macroscopic characteristics were used as pragmatic clinical indicators of colonization within the observational framework of this study. Due to the non-standardized and indication-based nature of microbiological testing, pathogen-specific analyses were not included in the primary outcome assessment.

Prior to enrolment, written informed consent was obtained from all patients after detailed information about the content and procedure of the study. All research was performed in accordance with relevant national and guidelines, regulations and in accordance with the declaration of Helsinki. Ethical approval was obtained from the responsible ethics committee of the Private University of Witten/Herdecke (responsible for studies conducted at Helios University Hospital Wuppertal – HUKW) before starting enrolment (UW/H No. 14/2018). Additional ethics approvals were obtained from the other participating study centers in Essen (18-8431-BO) and Duesseldorf (2020−1036). The study was registered in the German clinical trials register (DRKS) under DRKS00017384 and with the World Health Organization (WHO) under Universal Trial Number (UTN) U1111-1233-9350.

## pH and temperature measurements

The measurements were carried out using the NAWA Wound pH meter (TR26; Nawa Technology Limited, Nuremberg, Germany), an electric, mobile, CE-certified pH meter approved to measure the pH value and temperature in open wounds. The measurements were carried out according to a coordinated, predefined standard procedure to minimize measurement deviations between visits and centers due to differing procedures and applications. The pH meter was thoroughly cleansed and disinfected after each measurement using Mikrozid sensitive liquid (Schülke & Mayr GmbH, Norderstedt, Germany) as per manufacturers specifications and regularly calibrated (once per week).

The pH meter uses an ion-sensitive field-effect transistor (ISFET). This transistor can measure ionic concentrations. It consists of a channel made of semiconductor material (e.g. silicon). Instead of a classic metal gate electrode, the gate electrode is replaced by an ion-sensitive layer that comes into contact with the solution to be measured. A change in the pH value or the concentration of specific ions is measured by a change in the charge distribution on the surface of the ion-sensitive layer, as this influences the electrical state of the semiconductor channel. The surface charge of the ion-sensitive layer thus changes when the pH value (the concentration of hydrogen ions) changes. This change affects the channel conductivity of the field-effect transistor, which is reflected in a measured voltage.

Both the pH value and the temperature were measured at three different areas: wound center, wound edge (transition between the wound bed and surrounding skin) and immediate wound surrounding (≤ 5 cm from the wound edge). Thereby, any wound dressings were first removed. Prior to each measurement, the wounds were cleansed exclusively by mechanical debridement of loose adhering deposits, material residues and microbial deposits and rinsed or moistened with sterile 0.9% sodium chloride solution (NaCl). Antimicrobial/antiseptic irrigation or sharp or surgical debridement was not permitted prior to the measurements to prevent any influence on or induced change (e.g. due to bleeding, alkaline or acidotic rinsing solutions) in the pH value.

During measurements, the probe tip was carefully placed on the measurement spot at a 90° angle without applying pressure. After approx. 30 s the measured pH value and temperature appear on the display. The pH value and temperature are measured with a measurement accuracy of ± 0.2 for the pH value (measuring range: pH 3–12) and ± 0.5 °C for the temperature (measuring range: 15–40 °C).

### Statistical analysis

Data collection, storage, and processing were performed using Microsoft Excel (Microsoft Office) and statistical analysis of the data was performed using GraphPad Prism (version 10.2.1; GraphPad, Boston, MA, USA).

Continuous variables were presented as mean ± standard deviation (SD) or median and range, where appropriate, and categorical variables were presented as fractions and percentages.

Statistical analysis of the pH and temperature differences between the study arms with binary outcomes (e.g., acute versus chronic wounds, healing versus non-healing) was performed using an unpaired t-test for independent samples with Welch’s correction (for unequal variance). Further analyses of repeated measurements of pH values over the course of healing, as well as subgroup analyses to determine significant differences in pH values between multiple categories (e.g., different wound entities, different healing stages) were performed using analysis of variance (one-way ANOVA with Tukey post-hoc test). The accepted α error was set at 5%, resulting in a significance level of *p* < .05.

As part of the study planning, a case number calculation was performed in advance to determine the required number of patients. With a specified significance level α of 5% and an aspired power of 80%, based on results described in the literature to date and samples collected in smaller preliminary studies, at least 106 patients are required in total for binary outcome analyses (calculations based on unpaired t-test), so that, taking into account 10% oversizing, a total collective of 120 patients was determined. Due to planned multiple analyses, a Bonferroni correction of the significance level was performed in accordance with the expected analyses, which was included in the final case number calculation. The G*Power 3.1 program (Heinrich Heine University Duesseldorf) was used to calculate the number of cases.

## Results

### Demographic data

A total of 117 patients were included in the study. One or more follow-up measurements were performed on 55 patients. A total of 226 pH measurements and 181 temperature measurements were performed, 107 of which were part of the follow-up. Table 1 provides an overview of the key demographic data of the study participants included in the study for the entire group, differentiated according to acute and chronic wounds as well as healing and non-healing wounds.

**Table 1:** Demographic data of the study participants included in the study. Date is depicted stratified for the entire group, according to acute and chronic or healing and non-healing wounds.

Age (in years)	Total (*n* = 117)	Acute (*n* = 18)	Chronic (*n* = 99)	*p*-value	Healing (*n* = 62)	Non-healing (*n* = 55)	*p*-value
	70.70 (21–95)	66.73 (53–85)	71.46 (21–95)	0.241	69.60 (24–95)	72.10 (21–94)	0.376
Gender (n; %)				*0.253*			*0.768*
Male	57/117 (48.72)	11/18 (61.10)	46/99 (46.46)		31/62 (50.00)	26/55 (47.27)	
Female	60/117 (51.28)	7/18 (38.89)	53/99 (53.54)		31/62 (50.00)	29/55 (52.73)	
BMI	27.5 (13.3–44.1)	28.0 (18.3–37.6)	26.9 (13.3–44.1)	*0.567*	27.1 (17.4–44.1)	20.0 (13.3–38.9)	*0.939*
Nicotine consumption (n; %)				*0.148*			***< 0.0001***
*Smoking*	17/117 (14.50)	3/18 (16.67)	14/99 (14.14)		6/62 (9.68)	27/55 (49.09)	
*Non-smoking*	57/117 (48.7)	12/18 (66.67)	45/99 (45.45)		30/62 (48.39)	11/55 (20.00)	
*Unknown*	43/117 (36.8)	3/18 (16.67)	40/99 (40.40)		26/62 (41.94)	17/55 (30.90)	
Wound entity (n; %)				***0.004***			*0.209*
*DFU*	10/117 (8.5)	1/18 (5.56)	9/99 (9.09)		5/62 (8.06)	5/55 (9.09)	
*ALU*	40/117 (34.2)	7/18 (38.89)	33/99 (33.33)		18/62 (29.03)	22/55 (40.00)	
*VLU*	20/117 (17.0)	1/18 (5.56)	19/99 (19.2)		12/62 (19.35)	8/55 (14.55)	
*PU*	7/117 (6.0)	0/18 (0.0)	7/99 (7.07)		1/62 (1.62)	6/55 (10.91)	
*AVU*	12/117 (10.3)	1/18 (5.56)	11/99 (11.11)		7/62 (11.3)	5/55 (9.10)	
*WHD*	17/117 (14.5)	8/18 (44.44)	9/99 (9.01)		12/62 (19.35)	5/55 (9.10)	
*Others*	11/117 (9.4)	0/18 (0.0)	11/99 (11.11)		7/62 (11.3)	4/55 (7.27)	
Wound age *(in weeks)*	37.6 (1-364)	3.8 (1.0-7.5)	44.0 (21–95)	***0.011***	38.2 (1-288)	36.9 (1-364)	*0.912*

Values are given as mean and range or fractions of total and percentage. (DFU – diabetic foot ulcer, ALU – arterial leg ulcer, VLU – venous leg ulcer, PU – pressure ulcer, AVU – arterio-venous ulcer, WHD – wound healing disorder)

On average participants were 70.7 years (21–95) old. Acute wounds were present in 18 participants (15.38%) and chronic wounds in 99 participants (84.62%). Overall, there was no significant difference in age between the two subgroups (*p*=.241). There was also no significant difference in age between patients with healing or non-healing wounds (*p*=.376). The gender distribution showed a slightly higher proportion of female participants in the overall collective, at 51.28%, compared to 48.72% male participants, although the difference was not significant when comparing acute to chronic wounds *(p*=.253) or when comparing healing and non-healing wounds (*p*=.768).

In terms of the distribution of the wound entities examined, arterial leg ulcer (ALU) was the most common cause in 40 (34.2%) study participants. This was followed by venous leg ulcers (VLU) with a total of 20 (17.0%) cases. With 17 (14.5%) cases, postoperative wound healing disorders (WHD) were the third most common wound entity in this study. Arterio-venous ulcer (AVU) with twelve cases (10.3%), followed by other causes of wounds (“Other”) with eleven cases (9.4%), diabetic foot ulcer (DFU) with ten (8.5%) cases, and pressure ulcers (PU) in seven cases (6.0%) constituted the rest of the examined wounds. The average duration of the wounds was 37.6 (1–364) weeks for the entire group. Acute wounds existed for an average of 3.8 (1.0–7.5) weeks, whereas chronic wounds, by definition, existed for significantly longer, with an average of 44.0 (21–95) weeks. Wounds classified as healing at the time of assessment had existed for an average of 38.2 (1–288) weeks, while non-healing wounds had existed for an average of 36.9 (1–364) weeks.

Table 2 provides an overview of all pH measurements taken as part of the study (*n* = 226). The table shows the distribution of measurements across wound entities, types and stages examined and the average pH values in the wound center, edge and surrounding stratified by wound type (acute/chronic). In terms of clinical characteristics, 132 (58.41%) measurements were performed on wounds classified as “healing”, whereas 94 (41.59%) were performed on “non-healing” wounds. When considering the wound status, which was reclassified before each measurement, 96 (42.48%) of all measurements were performed during the granulation phase. This was followed by 86 (38.05%) measurements on wounds during the inflammation phase, 35 (15.49%) measurements during the epithelialization phase, and nine (3.98%) during a local infection. The degree of microbial burden of the wound was characterized as “not infected” in most of the measurements performed, namely 149 (65.93%). Fifty-six (24.78%) of all measurements were taken on colonized wounds, and 21 (9.29%) measurements were taken on infected wounds.

**Table 2:** Overview of all pH measurements collected as part of the study (*n* = 226). Demographics were stratified in acute and chronic wounds.

Wound entity (*n*; %)	Total (*n* = 226)	Acute (*n* = 39)	Chronic (*n* = 187)
*DFU*	14/226 (6.19)	3/39 (7.69)	11/187 (5.88)
*ALU*	94/226 (41.59)	12/39 (30.77)	82/187 (43.85)
*VLU*	32/226 (14.16)	1/39 (2.56)	31/187 (16.58)
*PU*	10/226 (4.42)	0/39 (0.0)	10/187 (5.35)
*AVU*	17/226 (7.52)	2/39 (5.12)	15/187 (8.02)
*WHD*	38/226 (16.81)	21/39 (53.85)	17/187 (9.09)
*Other*	19/226 (8.41)	0/39 (0.0)	19/187 (10.16)
Clinical tendency *(n; %)*			
*Healing*	132/226 (58.41)	25/39 (64.10)	107/187 (57.22)
*Non-healing*	94/226 (41.59)	14/39 (35.90)	80/187 (42.78)
Healing stage *(n; %)*			
*Infection*	9/226 (3.98)	1/39 (2.56)	8/187 (4.28)
*Inflammation*	86/226 (38.05)	12/39 (30.77)	74/187 (39.57)
*Granulation*	96/226 (42.48)	14/39 (35.90)	82/187 (43.85)
*Epithelialization*	35/226 (15.49)	12/39 (30.77)	23/187 (12.30)
Microbial burden *(n; %)*			
*Non-infected*	149/226 (65.93)	31/39 (79.49)	118/187 (63.10)
*Infected*	21/226 (9.29)	4/39 (10.27)	17/187 (9.09)
*Colonized*	56/226 (24.78)	4/39 (10.27)	52/187 (27.81)
pH value			
Wound center	6.91 (3.60–8.40)	6.96 (5.60–7.70)	6.96 (3.40–8.40)
Wound edge	7.00 (3.60–8.90)	6.78 (5.60–7.60)	7.07 (3.60–8.90)
Wound surrounding	6.76 (4.50–9.10)	6.72 (4.80–8.10)	6.77 (4.50–8.60)

Values are given as mean and range or fractions of total and percentage. (DFU – diabetic foot ulcer, ALU – arterial leg ulcer, VLU – venous leg ulcer, PU – pressure ulcer, AVU – arterio-venous ulcer, WHD – wound healing disorder)

Across all measured pH values (*n* = 226), the mean value was 6.91 (3.60–8.40) in the wound center, 7.00 (3.60–8.90) in the wound margin area, and 6.76 (4.50–9.10) in the wound surrounding. The mean pH value of all measurements in acute wounds was 6.96 (5.60–7.70) in the wound center, 6.78 (5.60–7.60) at the wound edge, and 6.72 (4.80–8.10) in the wound surrounding. In the chronic wound group, all pH values measured in the wound center area had a mean value of 6.96 (3.40–8.40), at the wound edge 7.07 (3.60–8.90), and in the wound surrounding 6.77 (4.50–8.60).

### Temperature and pH values in different wound entities

**Fig. 1:**
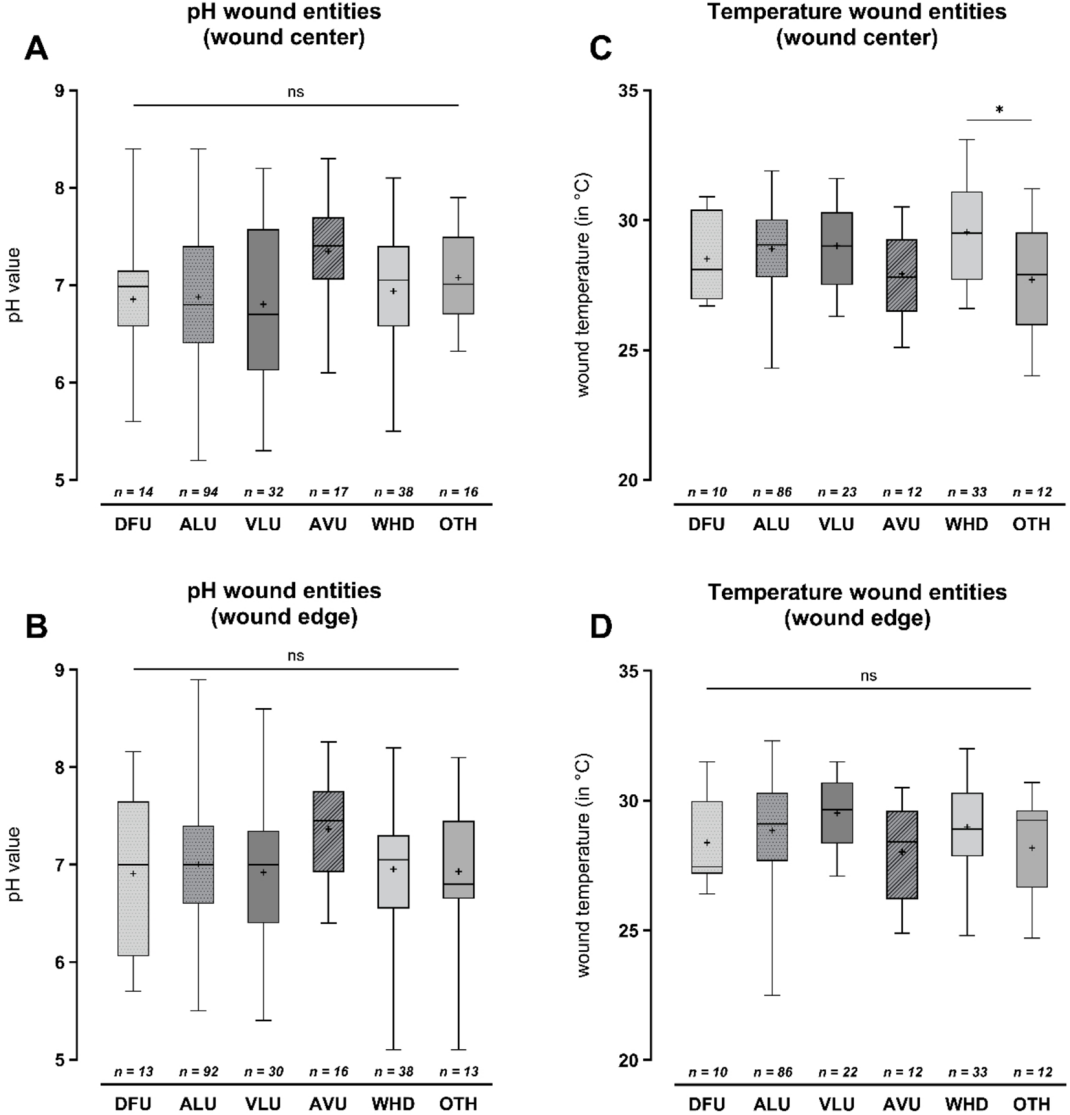
pH and temperature values measured in wound center (**A**, **C**) and edge (**B**, **D**) of different wound entities. The pH value (**A**, **B**) or temperature (**C**, **D**; in °C) is listed on the Y-axis. Results are shown as box plots (25%–75% percentile), the horizontal line marks the median, the “+” sign represents the mean value, and the error terms show the minimum and maximum. There was no significant difference between the wound entities examined in terms of the pH value in the wound center (*p *= .117) or wound edge (*p *= .344). Temperature measurements in WHD and OTH demonstrated a significantly higher temperature in WHD (*p *= .019) in the wound center but no significant difference in the wound edge (*p *= .713). Statistically significant differences are visualized by *ns* - not significant, *p < .05, **p < .01, ***p < .001, and ****p < .0001. DFU – diabetic foot ulcer; ALU – arterial leg ulcer; VLU –venous leg ulcer; AVU – arterio-venous ulcer; WHD – postoperative wound healing disorder; OTH – other wound entities (e.g., vasculitis).

Figure 1 shows the measured pH and temperature values of the wound entities included in the study in comparison with each other.

In the wound center, DFU showed a mean pH value of 6.855 ± 0.705 (5.60–8.40), while ALU showed a mean value of 6.877 ± 0.682 (5.20–8.40) and VLU a mean value of 6.805 ± 0.807 (5.30–8.20). The pH measurements in the group with AVU showed a higher mean value of 7.346 ± 0.591 (6.10–8.30) while WHD again demonstrated a lower mean value of 6.938 ± 0.666 (5.50–8.10). In the category “other” wound entities (OTHER), the mean value was 7.079 ± 0.473 (6.32–7.90). Overall, there was no significant difference in the pH value in the wound center when comparing the different entities (*p*=.117). The wound edge also showed no significant difference in pH values between different entities (*p*=.344). Mean values were 6.908 ± 0.831 (5.70–8.16) for DFU, 7.005 ± 0.650 (5.50–8.90) for ALU, 6.921 ± 0.807 (5.40–8.60) for VLU, 7.366 ± 0.532 (6.40–8.26) for AVU, 6.954 ± 0.570 (5.10–8.20) for WHD and 6.929 ± 0.789 (5.10–8.10) for “other” wound entities.

In terms of wound temperature in the wound center, a significant difference was only observed between WHD (29.54 ± 1.87 °C; range 26.60–33.10) and “other” wound entities (OTH; 27.70 ± 2.13 °C; range 24.00–31.20) with 1.90 ± 0.57 °C higher values in WHD (*p*=.019, 95%-CI [0.194; 3.479]). Comparisons between further entities showed no significant differences (*p*=.713). DFU demonstrated an overall mean temperature of 28.51 ± 1.66 °C (26.70–30.90), ALU of 28.90 ± 1.59 °C (24.30–31.90), VLU of 29.00 ± 1.51 °C (26.30–31.60) and AVU of 27.93 ± 1.76 °C (25.10–30.50).

## Differences in pH and temperature between acute and chronic wounds

**Fig. 2:**
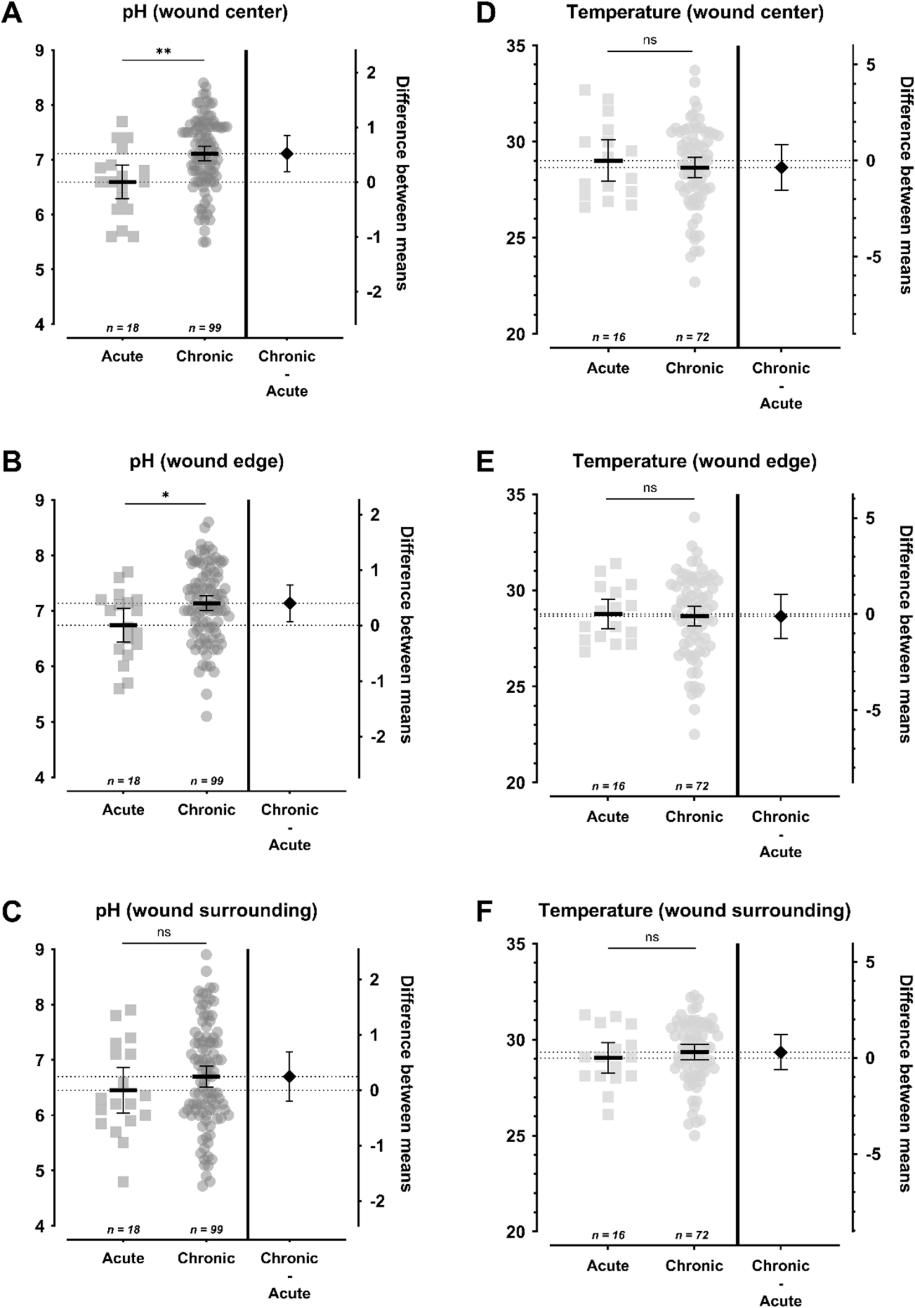
Comparison of pH (**A**-**C**) and temperature (**D**-**F**) measurements in acute vs. chronic wounds at the wound center (**A**, **D**), wound edge (**B**, **E**) and wound surrounding (**C**, **F**). The left Y-axis shows the pH value (**A**-**C**) or temperature (**D**-**F**; in °C), while the right Y-axis shows the difference between the compared mean values. The mean value is shown as a horizontal line with the 95% confidence interval as the error term. A significant difference in pH was observed between acute and chronic wounds in the wound center and wound edge, with higher values in chronic wounds (Δ_center_ = 0.521 ± 0.160, 95%-CI[0.192;0.850], *p *= .003; Δ_edge_ = 0.400 ± 0.160, 95%-CI[0.070;0.729], *p *= .020). No significant differences were observed for pH at the wound surrounding or for temperature measurements at any measurement area. Statistically significant differences are visualized by *ns* - not significant, *p<.05, **p<.01, ***p<.001, and ****p<.0001.

The comparison of pH values and temperature measured in the center, edge and surrounding of acute and chronic wounds is depicted in Fig. 2. The mean pH value in the wound center of acute wounds was significantly lower (Δ_center_ = 0.521 ± 0.160, *p*=.003, 95%-CI [0.192;0.850]) with 6.592 ± 0.617 (5.60–7.70) than in chronic wounds with 7.113 ± 0.659 (5.50–8.40). At the wound edge, the same significant difference could be observed with chronic wounds exhibiting a significantly higher mean pH of 7.136 ± 0.678 (5.10–8.60) compared to 6.736 ± 0.615 (5.60–7.70) in acute wounds (Δ_edge_ = 0.400 ± 0.160, *p*=.020, 95%-CI [0.070;0.729]). There was no significant difference between the pH values of acute and chronic wounds in the wound surrounding (Δ_surrounding_ = 0.248 ± 0.218, *p*=.265, 95%-CI [-0.200;0.696]). In acute wounds the mean pH was 6.450 ± 0.831 (4.80–7.90), while in chronic wounds, the mean value was 6.698 ± 0.944 (4.72–8.90).

Regarding the comparisons of temperature at the wound center, edge and surrounding, no significant differences could be detected between acute and chronic wounds. The mean value in the wound center in acute wounds was 29.01 ± 2.01 °C (26.60–32.70) compared to 28.65 ± 2.20 °C (22.70–33.70) in chronic wounds. At the wound edge, the mean temperature was 28.76 ± 1.46 °C (26.80–31.40) in acute wounds and 28.65 ± 2.18 °C (22.50–33.80) in chronic wounds, while at the surrounding the measurements were 29.04 ± 1.5 °C (26.10–31.30) in acute and 29.34 ± 1.69 °C (25.00-32.30) in chronic wounds.

## Differences in pH and temperature at different areas of wounds

**Fig. 3:**
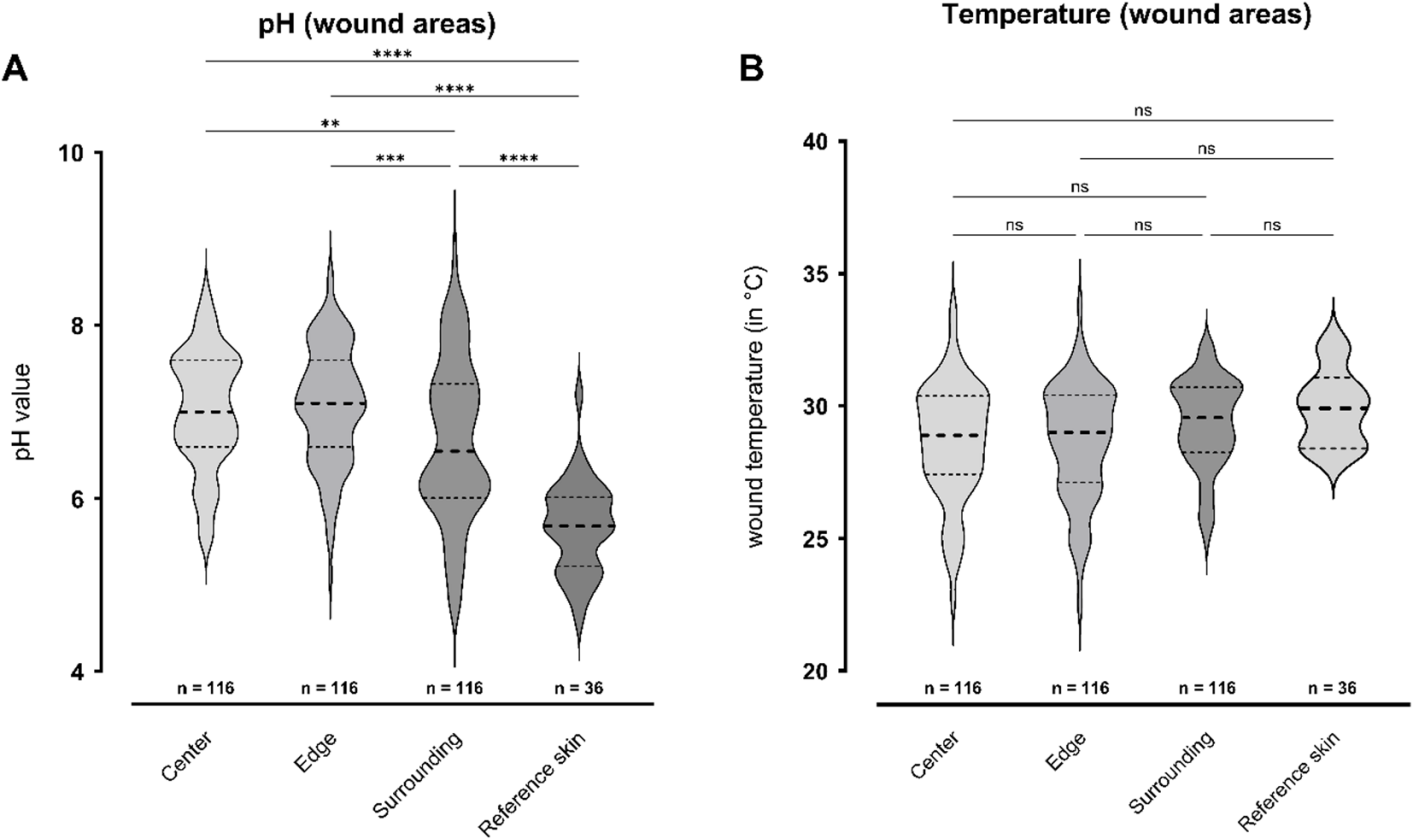
Comparison of pH (**A**) and temperature (**B**) measured in the wound center, wound edge, wound surroundings and a skin reference site on the contralateral, unaffected body site. The left Y-axis shows the pH value (**A**) or temperature (**B**; in °C). Results are shown as violin plots. The thicker dashed line indicates the median, while the finer dashed lines indicate the quartiles (25% and 75%). Significant differences between pH measurement sites were observed (*p*<.0001) with all measurements in the wound areas showing higher values than the reference skin. For the temperature, no significant differences were found between the various areas examined. Statistically significant differences are visualized by *ns* - not significant, *p<.05, **p<.01, ***p<.001, and ****p<.0001.

Different areas of a wound – center, edge and surrounding – were compared to a reference measurement on intact contralateral skin (Fig. 3). In the wound center, the mean value of all measured pH values was 7.032 ± 0.677 (5.50–8.40), while at the wound edge the pH was 7.074 ± 0.682 (5.10–8.60) showing no significant difference (Δ = 0.042, 95%-CI [-0.297;0.213]).*p*=.974). Comparing wound center pH to the surrounding area, a significant difference of 0.372 (95%-CI [0.117;0.672], *p*=.001) was observed with a mean value of 6.660 ± 0.929 (4.72–8.90) in the wound surrounding. The reference skin also showed a significantly lower mean pH of 5.670 ± 0.527 (4.61–7.20) compared to the center (Δ = 1.361, 95%-CI [0.991;1.732], *p*<.0001).

When comparing the pH at the wound edge to the wound surrounding (Δ = 0.414, 95%-CI [0.159;0.669], *p*=.0002) and to the reference skin (Δ = 1.403, 95%-CI [1.033;1.774], *p*<.0001), measurements at the wound edge were significantly higher in both cases. Generally, the reference skin showed significantly lower pH values compared to all other areas, including the wound surrounding (Δ = 0.989, 95%-CI [0.619;1.360], *p*<.0001).

In terms of the temperature, the mean value of all measured values in the wound center was 28.65 ± 2.20 °C (22.70–33.70). The average value at the wound edge was 28.65 ± 2.20 °C (22.50–33.80). In the area surrounding the wound, the average value was 29.34 ± 1.69 °C (25.00-32.30). The reference skin showed an average temperature of 29.92 ± 1.46 °C (28.40–32.20). There was no significant difference between the different measurement locations.

### Comparison of pH and temperature between healing and non-healing wounds

**Fig. 4:**
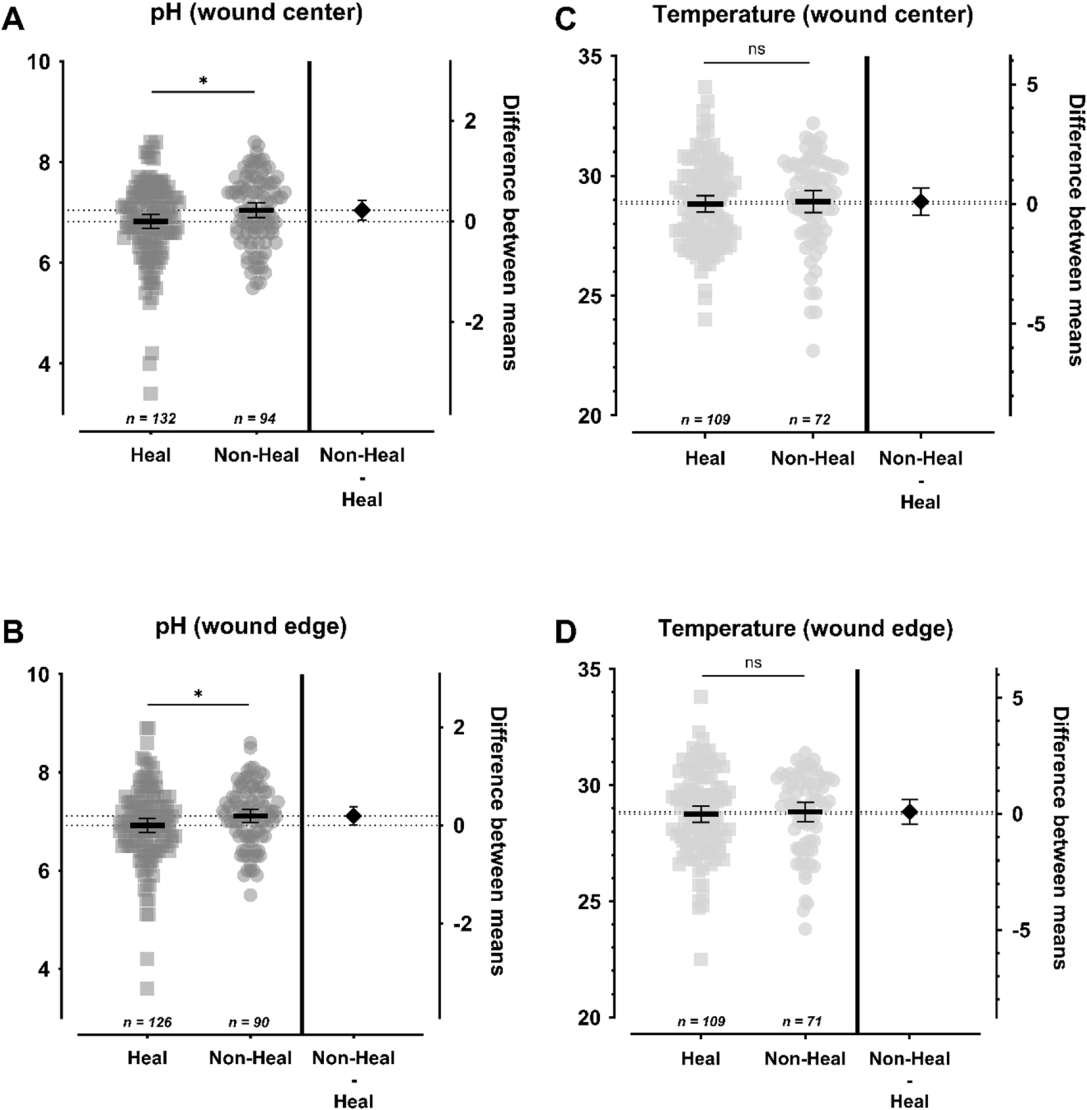
C, DComparison of pH (**A**, **B**) and temperature (**C, D**) measured in the wound center and wound edge of healing vs. non-healing wounds. The left Y-axis shows the pH value (**A, B**) or temperature (**C**, **D**; in °C), while the right Y-axis shows the difference between the compared mean values. The mean value is shown as a horizontal line with the 95% confidence interval as the error term. A significant difference in pH was observed between healing and non-healing wounds in the wound center and wound edge, with higher values in non-healing wounds (Δ_center_ = 0.223 ± 0.101, *p*=.028, 95%-CI[0.024;0.421]; Δ_edge_ = 0.192 ± 0.097, *p*=.049, 95%-CI[0.0009;0.384]). No significant differences were observed regarding temperature measurements. Statistically significant differences are visualized by *ns* - not significant, *p<.05, **p<.01, ***p<.001, and ****p<.0001.Julian-Dario Rembe1,*,#, Mareike WitteC, DC, DC, DC, D

Figure 4 shows the average pH values and temperature measurements in the center and edge of healing wounds compared to non-healing wounds.

The group of healing wounds had a lower mean pH value of 6.820 ± 0.814 (3.40–8.40) in the center as compared to the non-healing wounds with 7.043 ± 0.694 (5.50–8.40), which proved significantly different (Δ = 0.223 ± 0.101, *p *= .028, 95%-CI [0.024;0.421]). At the wound edge healing wounds also had a significantly lower pH (Δ = 0.192 ± 0.097, *p *= .049, 95%-CI [0.001; 0.384]). Healing wounds showed a mean value of 6.920 ± 0.803 (3.60–8.90) compared to non-healing wounds with 7.112 ± 0.623 (5.50–8.60).

Regarding temperature measurements, no significant difference was found between healing and non-healing wounds. Healing wounds had a mean temperature of 28.82 ± 1.81 °C (24.00-33.70) at the center and 28.76 ± 1.85 °C (22.50–33.80) at the edge. Non-healing wounds had a mean temperature of 28.92 ± 1.93 °C (22.70–32.20) at the center and 28.85 ± 1.73 °C (23.80–31.40) at the edge.

### Comparison of pH and temperature between colonized and non-colonized wounds

**Fig. 5:**
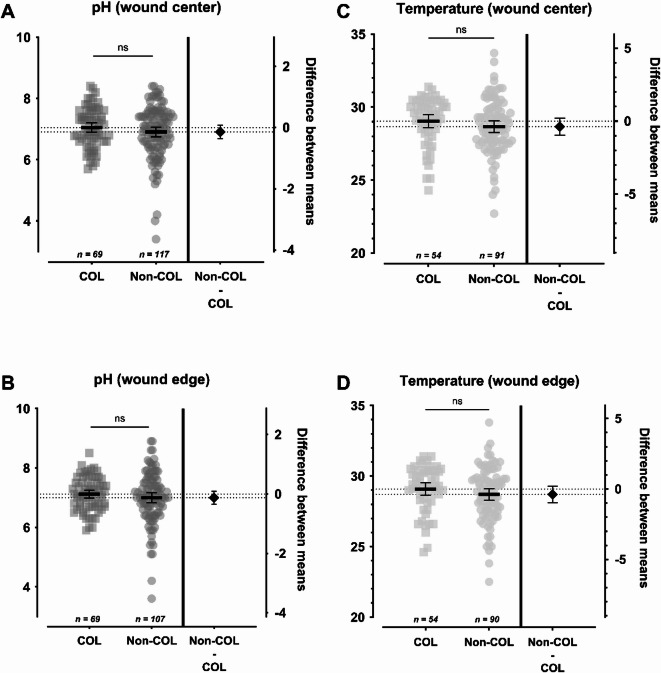
Comparison of pH (**A**, **B**) and temperature (**C**, **D**) measured in the wound center and wound edge of colonized vs. non-colonized wounds. The left Y-axis shows the pH value (**A**, **B**) or temperature (**C**, **D**; in °C), while the right Y-axis shows the difference between the compared mean values. The mean value is shown as a horizontal line with the 95% confidence interval as the error term. No significant difference in pH or temperatures was observed between colonized and non-colonized wounds in the wound center or wound edge. Statistically significant differences are visualized by *ns* - not significant, *p<.05, **p<.01, ***p<.001, and ****p<.0001.

The average pH values and temperature measurements in the center and edge of colonized wounds compared to non-colonized wounds is depicted in Fig. 5. Neither pH nor temperature measurements showed any significant differences between colonized and non-colonized wounds.

Table 3 depicts a summary of microbiological wound swab results, where performed when clinically indicated as part of routine care. Among all 226 consultations microbiological diagnostics was initiated in 11.1% of cases, of which 44% yielded positive cultures. The most frequently isolated pathogens were *Staphylococcus aureus* (SA), *Pseudomonas aeruginosa* (PA) and *E. coli* (EC), accounting for 54.6% for SA and 27.3% for PA and EC of positive cultures. All wounds receiving microbiological swabs were also classified as either infected (*n* = 21) or colonized (*n* = 4). Due to the indication-based and non-standardized nature of microbiological testing, these findings are descriptive and were not included in inferential analyses.

**Table 3:** Overview of microbiological diagnostics results. No systematic microbiological study-associated diagnostics were conducted, only in clinically indicated cases. These cases and thereby identified species are displayed (in some cases multiple species were identified in one swab culture).

Culture initiated (*n*, %)	Total (*n* = 226)
	25/226 (11.1)
*Positive*	11/25 (44.0)
*Negative*	14/25 (56.0)
Microorganisms identified	
*Staphylococcus aureus*	6/11 (54.6)
*Pseudomonas aeruginosa*	3/11 (27.3)
*Escherichia coli*	3/11 (27.3)
*Serratia liquetacies*	1/11 (9.1)
*Streptococcus dysgalacticae*	1/11 (9.1)
*Acinetobacter baumanii*	1/11 (9.1)
*Corynebakterien*	1/11 (9.1)
*Enterococcus faecalis*	1/11 (9.1)
*Klebsiella pneumoniae*	1/11 (9.1)

Colonized wounds showed a pH in the wound center of 7.047 ± 0.644 (5.70–8.40) and at the wound edge of 7.123 ± 0.558 (5.90–8.50). Non-colonized wounds demonstrated a pH value of 6.903 ± 0.877 (3.40–8.40) in the center and 6.996 ± 0.881 (3.60–8.90) at the edge. The temperature measurements showed 29.04 ± 1.66 °C (24.30–31.40) at the wound center for colonized wounds and 28.66 ± 1.90 °C (22.70–33.70) for non-colonized wounds. At the wound edge the temperature values were 29.08 ± 1.61 °C (24.60–31.40) in colonized and 28.69 ± 1.90 °C (22.50–33.80) in non-colonized wounds.

### pH values and temperature measurements depending on the wound healing phase

**Fig. 6:**
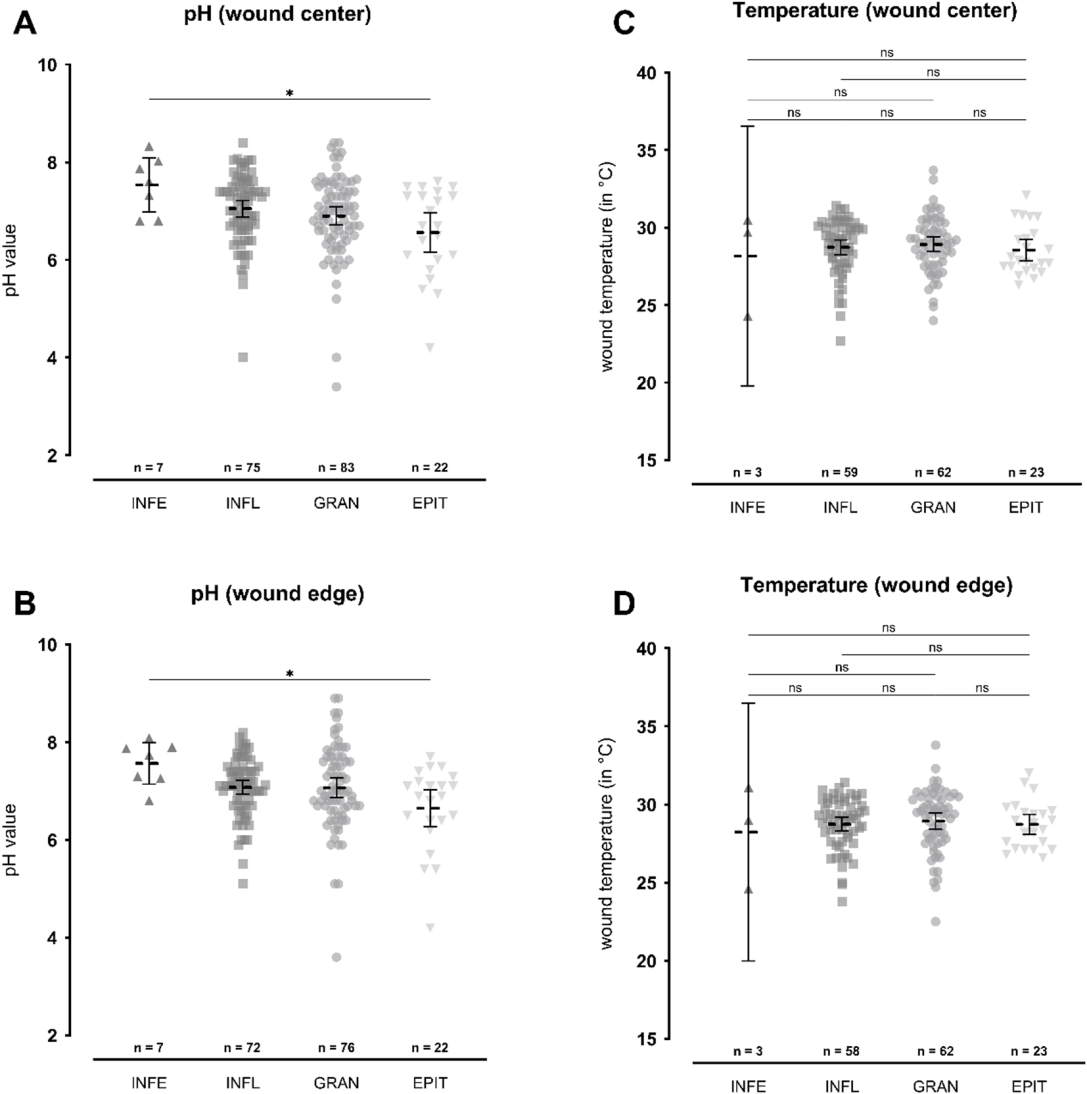
Comparison of pH (**A,** **B**) and temperature (**C**, **D**) measured during wound healing phases infection (INFE), inflammation (INFL), granulation (GRAN) or epithelization (EPIT). The left Y-axis shows the pH value measured at the wound center (**A**) or edge (**B**) and the temperature (in °C) measured at the wound center (**C**) or edge (**D**). Results are shown as scatter plots. The mean value is shown as a horizontal dashed line with the 95% confidence interval as the error term. Overall, there is a significant difference between the wound healing phases in terms of pH value (*p*=.018). A higher pH was observed in the wound infection group compared to wounds in the epithelialization phase, both in the center (Δ = 0.971; 95%-CI [0.067; 1.874], *p*=.030) and at the wound edge (Δ = 0.916; 95%-CI [0.055; 1.777], *p*=.032). No significant differences were observed in temperature measurements. Statistically significant differences are visualized by *ns* - not significant, *p<.05, **p<.01, ***p<.001, and ****p<.0001.

Figure 6 shows the comparison of the pH and temperature values in the center and edge of wounds in the various phases of wound healing; Table 4 summarizes the mean values measured.

**Table 4:** pH and temperature measurements stratified by wound healing phase at the wound center and wound edge.

	pH			Temperature (in °C)		
	Mean	SD	Range	Mean	SD	Range
Center						
*Infection*	7.534	0.593	6.80–8.33	28.17	3.37	24.30–30.50
*Inflammation*	7.050	0.731	4.00-8.40	28.73	1.82	22.70–31.40
*Granulation*	6.899	0.847	3.40–8.40	28.92	1.86	24.00-33.70
*Epithelization*	6.564	0.913	4.20–7.60	28.54	1.59	26.30–32.10
Edge						
*Infection*	7.566	0.458	6.80–8.09	28.23	3.32	24.60–31.10
*Inflammation*	7.076	0.613	5.10–8.20	28.74	1.68	23.80–31.40
*Granulation*	7.068	0.880	3.60–8.90	28.95	2.03	22.50–33.80
*Epithelization*	6.650	0.851	4.20–7.70	28.73	1.50	26.60–32.00

Overall, there was a significant difference in pH values between the healing phases (*p*=.018). When comparing the respective phases with each other, infected wounds showed significantly higher pH levels than the wounds undergoing epithelialization (Δ = 0.971, 95%-CI [0.067;1.874], *p *= .030). No significant differences were found when comparing the other phases with each other. At the wound edge, there was also a significant overall difference observed (*p *= .028). Comparing the respective phases separately revealed a significantly higher pH in infected wounds compared to wounds in the epithelialization phase (Δ = 0.916, 95%-CI [0.055;1.777], *p *= .032). No significant differences were found when comparing the other phases with each other at the wound edge.

In terms of the temperature values at the wound center, there was no significant difference between the healing phases of the wounds examined (*p *= .767). For values at the wound edge, there was also no significant difference observed between the healing phases (*p *= .854).

### Measurements of pH and temperature over the course of wound healing

**Fig. 7:**
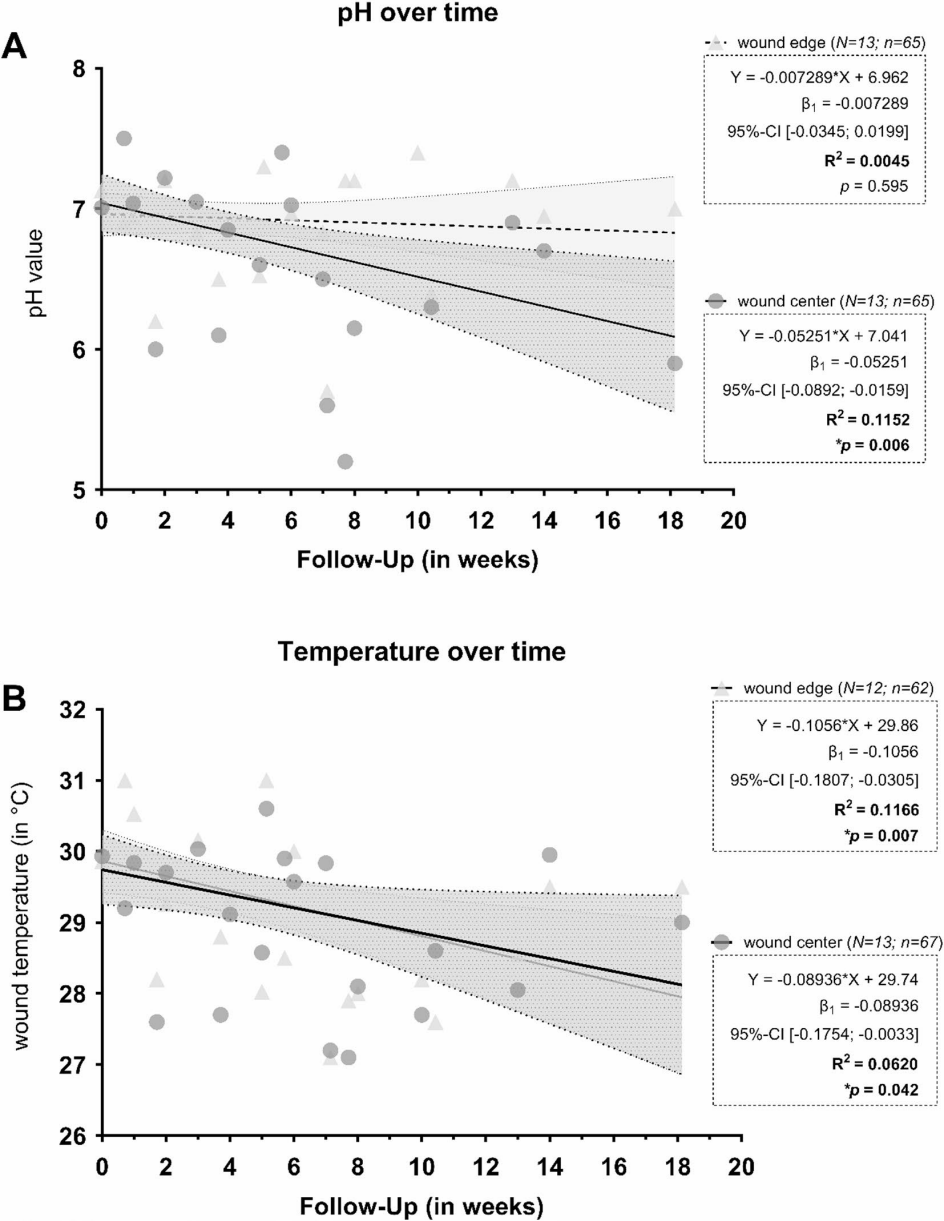
Representation of the pH (**A**) and temperature (**B**) values of the follow-up measurements in the wound center and wound edge using adjusted trend lines. The pH (**A**) or temperature (**B**) value is shown on the Y-axis, while the X-axis depicts the time of the follow-up measurements in weeks. The individual measurements are shown as points (circles – wound center; triangles – wound edge), as well as trend lines adjusted by linear regression (solid line – wound center; dashed line – wound edge) and associated error bands (shaded areas; dark gray – wound center, light gray – wound edge). Both measurement areas show a decreasing trend in pH and temperature over time. For pH the wound center showed a significant divergence (decrease) from zero (*p *= .006) and a greater decrease over time compared to the pH value at the wound edge (*p *= .0495). The pH value in the wound center decreases by 0.053 units per week over time. For the temperature both, center and edge, showed a decreasing trend over time, with both displaying a significant divergence (decrease) from zero (*p *= .042 and *p *= .007). Comparing measurements in the center and at the edge revealed no significant difference (*p *= .777). The temperature in the wound center decreases by 0.09 °C per week and at the wound edge by 0.11 °C. The equation of the adjusted straight line with the regression coefficient β1 and its 95% confidence interval and the coefficient of determination (R^2^) are also shown.

**Fig. 8:**
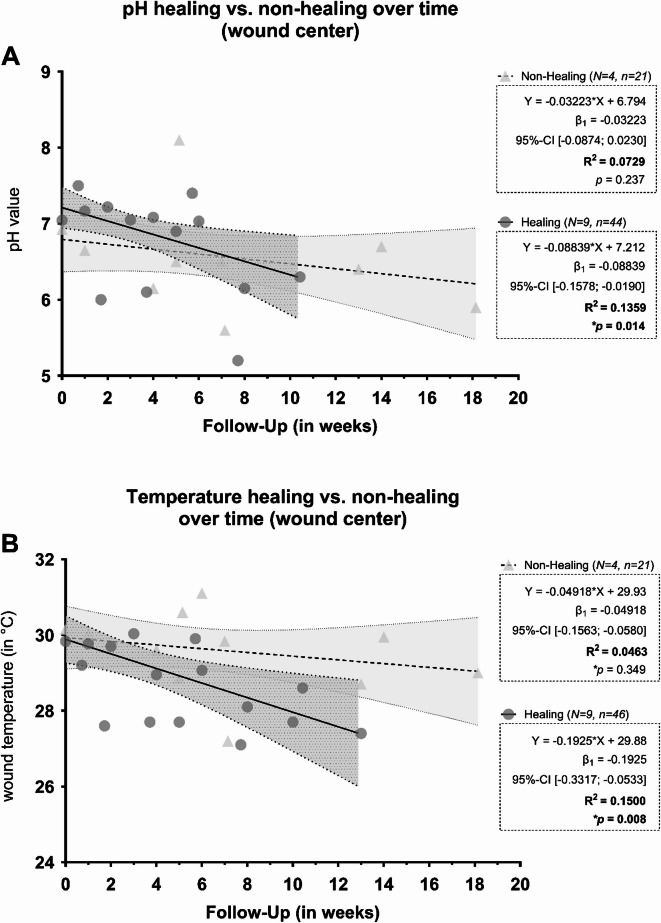
Representation of the pH (**A**) and temperature (**B**) values of the follow-up measurements in healing vs. non-healing wounds using adjusted trend lines. The pH (**A**) or temperature (**B**) value is shown on the Y-axis, while the X-axis depicts the time of the follow-up measurements in weeks. The individual measurements are shown as points (circles – healing; triangles –non-healing), as well as trend lines adjusted by linear regression (solid line – healing; dashed line – non-healing) and associated error bands (shaded areas; dark gray – healing, light gray – non-healing). Both measurement ranges show a decreasing trend in pH over time, with only healing wounds showing a significant divergence (decrease) from zero (*p *= .014). The pH value of non-healing wounds shows no significant divergence (*p *= .237) and overall, there is no significant difference between the lines for healing and non-healing wounds (*p *= .199). The pH value in the center of healing wounds decreases by 0.088 units per week of healing. For temperature measurements (B), both ranges show a decreasing trend over time, with only healing wounds showing a significant divergence (decrease) from zero (*p *= .008). The temperature of non-healing wounds shows no significant divergence (*p *= .349) and overall, there is no significant difference between healing and non-healing wounds (*p *= .110). The wound temperature in the center of healing wounds decreases by 0.19 °C per week of healing. The equation of the adjusted straight line with the regression coefficient β1 and its 95% confidence interval and the coefficient of determination (R^2^) are also shown.

The results for pH and temperature in the wound center and at the wound edge as well as comparing healing and non-healing wounds over the treatment course are depicted in Figs. 7 and 8. All patients with chronic wounds, for which at least four measurements per wound were available were included in the longitudinal analyses. The follow-up measurement period extended to up to 127 days after recruitment.

The analysis was performed using linear regression, revealing a general downward trend for pH values over the course of the observation period. In the wound center, pH decreased significantly more over time than at the wound edge (*p *= .0495) and showed a significantly steeper decrease (*p *= .006). This resulted in a reduction in pH of 0.053 points per week in the wound center (95%-CI [− 0.089; − 0.016]). However, the coefficient of determination for the adjustment of data using linear regression was only R^2^ = 0.1152, meaning that approximately 11.5% of the data can be explained by the model. The temperature measurements also showed a general downward trend over the course of the observation period. However, there was no significant difference between the reduction in temperature when comparing the wound center and the wound edge (*p*=.777). Both areas (wound center and wound edge) showed a significant deviation from zero with a decrease in temperature during the follow-up period (*p*=.042 and *p*=.007). This resulted in a weekly reduction in temperature of 0.09 °C in the wound center (95%-CI [− 0.175; − 0.003]) and 0.11 °C at the wound edge (95% CI [− 0.181; − 0.031]). The coefficient of determination yielded an R^2^ of 0.1166 for the wound edge and 0.0620 for the wound center, meaning that approximately 11.7% and 6.2% of the data, respectively, can be explained by the model.

Comparing healing and non-healing wounds (Fig. 8), the pH value also shows a decreasing trend for both stages in general. However, healing wounds show a steeper decrease in the wound center, with a reduction in pH of 0.088 (95%-CI [− 0.158; − 0.019]) per week of progressive healing. This reduction is significantly different from zero (*p*=.014), but not significantly different compared to non-healing wounds (*p*=.199). The decrease in pH non-healing wounds itself does not represent a significant deviation from zero (*p*=.237). A comparison of wound temperature between healing and non-healing wounds also shows an overall decreasing trend. Here, too, healing wounds show a steeper decrease in temperature, with a reduction of 0.19 °C (95%-CI [− 0.332; − 0.053]) per week. This reduction is significantly different from zero (*p*=.008), but not significantly different from non-healing wounds (*p*=.110).

## Discussion

This prospective observational study examined pH and temperature as local biomarkers in acute and chronic wounds across various wound types, healing tendency, and clinical classifications. The study confirms the pivotal role of pH and temperature as physiochemical parameters correlating with wound healing. The findings support and expand upon the growing body of evidence suggesting that a lower, more acidic pH is associated with healing wounds, while alkaline values tend to correlate with chronicity, microbial burden, and stagnation of the healing process^1,7,16,21,22^.

Our results demonstrate significant spatial pH differences within the wound environment. The wound bed and edge displayed markedly higher pH values compared to intact peripheral skin, reflecting the loss of the skin’s natural acid mantle and exposure of subepidermal tissue with near-neutral pH values (~ 7.4; Fig. 3A)^9,10,22^. The average pH difference of 1.3 units between wound center and healthy skin highlights the extent of this disruption. A key observation was the gradient of decreasing pH from wound center toward the periphery, suggesting a physiological shift toward re-acidification as epithelialization progresses. This trend was consistent with clinical wound healing stages, where wounds in the infection or inflammation phase had significantly higher pH values than those in granulation or epithelialization (*p*=.03; Fig. 6A,B), with the lowest pH levels observed in healing wounds (Fig. 4A,B). This pattern was evident across all measured sites (wound bed, wound edge and wound surrounding), indicating that acidification accompanies and potentially facilitates tissue regeneration. These findings align with previous studies reporting a similar association between acidic pH and healing. Shukla et al.^23^, Kaufmann et al.^24^, and Wiegand et al.^25^ have shown that acidic environments promote epithelialization, collagen synthesis, and angiogenesis in both clinical and animal models. However, a recent review by Derwin et al.^5^ emphasized the lack of high-quality interventional data supporting pH-targeting therapies, reinforcing the need for further trials.

The comparison between acute and chronic wounds confirmed significantly higher pH values in chronic wounds, particularly at the wound center (Δ = 0.52; *p*=.003; Fig. 2A) and edge (Δ = 0.40; *p*=.02; Fig. 2B). Similarly, healing wounds exhibited lower pH values than non-healing wounds (Δ = 0.22; *p*=.03; Fig. 4A,B). These findings align with other reports that healing wounds tend to have more acidic environments, while stagnant or infected wounds are more alkaline^5,6^ and reinforce the hypothesis that chronic wounds are characterized by a shift toward alkalinity, potentially driven by impaired barrier function, persistent inflammation, and bacterial burden and metabolism^8,14,21^. Additionally, the exposure of subcutaneous tissue with the above-described alkaline environment and wound exudate can further impair the skin barrier function surrounding the wound. Thereby excessive or poorly managed exudate from the wound bed raises the pH level of the surrounding acid mantle, disrupting the local microbiome and further jeopardizing the skin barrier function^26^, in turn leading to a potential increase in wound size. The significantly higher pH value compared to the reference skin on the contralateral side reflects current findings on wound area cleansing and care^27^. These findings recommend including the surrounding skin up to 20 cm from the wound edge at each dressing change^28^.

Interestingly, our data showed a narrower pH range within the wound than previously reported^5,29,30^, with most values clustering around pH 7.0 and an overall range of 5.1–8.6. This tighter distribution may reflect the use of a more precise measurement method (ISFET-based pH microprobe), in contrast to pH indicator strips used in some earlier studies^5^. It also suggests that even modest shifts in pH—on the order of 0.2 to 0.5 units—may carry pathologic as well as diagnostic or prognostic value, mirroring the tight physiological pH regulation seen in human blood (7.35–7.45)^22^. In this context, although pH variation in our cohort was moderate compared to previous studies, its predictive value for healing appears meaningful. Given the physiological importance of tight pH regulation, even subtle shifts may reflect or influence cellular activity. This parallels blood pH homeostasis, where deviations of just 0.1 units can have systemic consequences and may necessitate clinical correction^22^.

A longitudinal subset analysis revealed a consistent weekly decrease in pH during wound healing (0.05 pH units/week, *p*=.006; Fig. 7A), with healing wounds showing a steeper decline (0.09 units/week) compared to non-healing wounds (0.03 units/week; Fig. 8A). Although the predictive strength (R^2^=0.13) was modest, the pattern reinforces the potential utility of pH monitoring as a biomarker of healing progression. The consistent association of healing with pH reduction opens avenues for therapeutic pH modulation. Experimental studies have shown that local acidification, e.g. by using citric or phosphoric acid, can accelerate epithelialization and collagen synthesis^15,16^. However, systematic reviews emphasize the lack of sufficient evidence to recommend specific acidifying products^5,6^. Our findings support the rationale for future interventional studies using acidifying dressings or solutions, provided that such interventions are standardized and monitored.

Microbial colonization was associated with higher pH values, although not statistically significant in our cohort (Fig. 5A,B). This may be due to the outpatient recruitment setting, where severely infected wounds have already been treated in-hospital. However, wounds classified as clinically infected during measurement did exhibit the highest pH levels, supporting prior evidence that bacterial metabolism contributes to alkalization^31^. The highest average pH values were found in arterio-venous ulcers (Fig. 1A,B), potentially reflecting the cumulative effects of chronic inflammation, proteolysis, hypoxia, and microbial load. Hypoxic tissue breakdown, cellular lysis, and bacterial metabolism release basic compounds such as ammonia, bicarbonate, and amines, which contribute to local alkalization. This supports prior work describing alkaline wound beds as more susceptible to infection and delayed healing^8,14,21,31^.

Temperature measurements across wound areas revealed smaller differences between acute and chronic wounds or healing and non-healing wounds than pH (Figs. 2D-E and 4C,D). Only wound healing disorders (WHD) had significantly higher average temperatures (*p*=.019). However, a longitudinal decrease in wound bed temperature was observed over the treatment period (0.09–0.10 °C/week, *p*=.042), with healing wounds again showing a more pronounced decline (0.19 °C/week, *p*=.008) than non-healing wounds (0.05 °C/week, *p*=.349). These results align with previous reports of temperature decline as a marker of healing^5,32^. Interestingly, while pH and temperature are known to interact physiochemically, higher temperature typically lowering pH, no such inverse relationship was observed. Instead, both parameters trended downward in healing wounds, suggesting a complex but parallel association with wound resolution (Figs. 7B and 8B) .

To the best of our knowledge, with 117 patients and 226 measurements, this prospective, observational multi-center study is among the largest of its kind to analyze pH and temperature in patients with acute and chronic wounds. However, limitations include a smaller sample of acute wounds and longitudinal follow-up, lack of standardization in environmental temperature and potential variability in local therapy. Additionally, clinical staging of wounds was based on subjective expert assessment, though this reflects real-world conditions. This aspect also applies for the absence of standardized microbiological testing within the study protocol. Microbiological swabs were performed only when clinically indicated as part of routine care and colonization status was therefore determined primarily by clinical assessment rather than systematic pathogen identification. Consequently, pathogen-specific analyses and their potential association with pH or temperature profiles could not be evaluated. Future studies should therefore incorporate additional validated classification systems and objective outcome measures. Despite these constraints, the use of a calibrated, contact-based pH probe offers improved precision over traditional methods^5,22^, and the prospective design with a real-world patient cohort enhances the reliability of the associations observed.

Our study confirms that wound pH decreases with healing progression and is significantly lower in acute and healing wounds compared to chronic and non-healing wounds. The trend of acidification from wound center to periphery, and over time, may reflect restoration of the physiological skin barrier and tissue homeostasis. Temperature also declines during healing, though its diagnostic utility appears more limited. Together, these findings suggest that pH, and to a lesser extent temperature, could serve as valuable, non-invasive biomarkers for monitoring wound status. They also encourage interventional studies which should evaluate whether targeted acidification of chronic wounds can actively promote healing, as suggested by early animal data^16^. Further research should clarify the causal interplay between pH, temperature, microbial load, and host immune response, ideally in stratified cohorts and controlled clinical settings, and define actionable thresholds for clinical decision-making. In the future, these findings may contribute to more precise, biomarker-guided wound, individual problem-oriented management strategies.

## Data Availability

The datasets generated and/or analyzed during the current study are available from the corresponding author on reasonable request.
